# Fiducial Markers Allow Accurate and Reproducible Delivery of Liver Stereotactic Body Radiation Therapy

**DOI:** 10.3390/curroncol30050382

**Published:** 2023-05-16

**Authors:** Marina Moskalenko, Bernard L. Jones, Adam Mueller, Shirley Lewis, Jay C. Shiao, Sara J. Zakem, Tyler P. Robin, Karyn A. Goodman

**Affiliations:** 1 Department of Radiation Oncology, University of Colorado School of Medicine, Aurora, CO 80045, USA; 2 Department of Radiation Oncology, Thomas Jefferson University, Philadelphia, PA 19107, USA; 3 Department of Radiotherapy and Oncology, Kasturba Medical College, Manipal Academy of Higher Education, Manipal 576104, India; 4 Department of Radiation Oncology, University of Washington, Seattle, WA 98195, USA; 5 Department of Radiation Oncology, Icahn School of Medicine at Mount Sinai, New York, NY 10029, USA

**Keywords:** fiducial marker, liver tumor, SBRT, IGRT, radiation

## Abstract

Fiducial markers are utilized for image guided radiotherapy (IGRT) alignment during the delivery of liver stereotactic body radiosurgery (SBRT). There are limited data demonstrating the impact of matching fiducials on the accuracy of liver SBRT. This study quantifies the benefit of fiducial-based alignment and improvements in inter-observer reliability. Nineteen patients with 24 liver lesions were treated with SBRT. Target localization was performed using fiducial markers on cone-beam computed tomography (CBCT). Each CBCT procedure was retrospectively realigned to match both the liver edge and fiducial markers. The shifts were recorded by seven independent observers. Inter-observer variability was analyzed by calculating the mean error and uncertainty for the set-up. The mean absolute Cartesian error observed from fiducial and liver edge-based alignment was 1.5 mm and 5.3 mm, respectively. The mean uncertainty from fiducial and liver edge-based alignment was 1.8 mm and 4.5 mm, respectively. An error of 5 mm or greater was observed 50% of the time when aligning to the liver surface versus 5% of the time when aligning to fiducial markers. Aligning to the liver edge significantly increased the error, resulting in increased shifts when compared to alignment to fiducials. Tumors of 3 cm or farther from the liver dome had higher mean errors when aligned without fiducials (4.8 cm vs. 4.4 cm, *p* = 0.003). Our data support the use of fiducial markers for safer and more accurate liver SBRT.

## 1. Introduction

Stereotactic body radiotherapy (SBRT) delivers conformal high-dose radiation and is used as focal liver therapy in the management of primary liver cancers and oligometastatic lesions with excellent local control rates of >80% [1,2,3,4]. Given the high doses administered per fraction, a reduction in liver motion using respiratory gating, abdominal compression, or deep-inspiration breath hold is important for improving treatment accuracy. Precise tumor localization is paramount in order to reduce uncertainties in radiation delivery and minimize radiation-induced liver toxicity particularly in the era of new targeted drug therapies whereby interaction with SBRT may be unknown. However, it can be challenging to identify liver tumors on cone-beam computed tomography (CBCT) scans used for image-guided radiation therapy (IGRT) prior to SBRT treatment [5].

The utilization of fiducial markers for IGRT alignment during SBRT has been shown to be an effective radio-opaque surrogate for tumor position [5]. Treatment set-up using marker guidance for liver SBRT has demonstrated reductions in set-up error when compared to conventional set-up methods including no correction, alignment to vertebrae, and 3D diaphragm-based set-up [6]. However, the additional procedure of implanting fiducial markers is logistically cumbersome and not without risk. Since treatment precision is associated with marker–tumor distance, substantial errors might result if fiducial markers are not implanted precisely [7,8].

Limited data exist comparing liver edge versus fiducial markers as a surrogate for tumor localization, when evaluating the accuracy of liver SBRT delivery. In this study, we aim to define the benefit of fiducial-based alignment and analyze inter-observer reliability in target alignment for liver SBRT.

## 2. Materials and Methods

Between November 2015 and May 2018, nineteen patients with 24 liver tumors were treated with SBRT on a TrueBeamSTX linear accelerator (Varian Medical Systems, Palo Alto, CA, USA) at a single institution. Prior to treatment, 1–4 gold fiducial markers (Alpha-Omega Services, Inc., Bellflower, CA, USA, Ref. # SMG0242-02) were implanted by an interventional radiologist in close proximity to the tumor under computed tomography (CT) guidance approximately 1 week prior to treatment. A planning CT scan with intravenous contrast was performed followed by a four-dimensional (4D) CT scan to assess organ motion. To minimize motion, all patients were planned for using either abdominal compression or amplitude-based respiratory gating at the discretion of the treating physician. The gross tumor volume (GTV) was defined using the planning CT or a magnetic resonance imaging (MRI) scan. When feasible, an internal target volume (ITV) was drawn to include the respiratory-related motion of the tumor and a uniform expansion of 5 mm was utilized to create the planning target volume (PTV). Treatment planning was completed in Eclipse using volumetric modulated arc therapy (Varian Medical Systems, Palo Alto, CA, USA). Prior to treatment, a CBCT scan was acquired and aligned to the planning CT using the fiducials as a guide. All data were collected under an institutional review board-approved protocol for retrospective data analysis.

**Figure 1:**
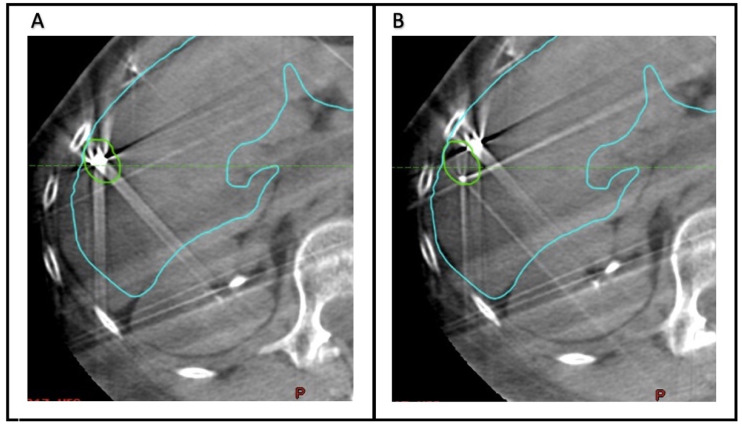
Comparison of retrospective offline alignment using fiducials versus liver-edge. (**A**) Fiducial-based alignment. the fiducial contour plus 2mm margin (green contour) was used as a source for alignment, demonstrating posterior–lateral liver edge misalignment (cyan contour). (**B**) Liver-edge alignment: the posterior–lateral liver edge (cyan contour) was used as a source for alignment, demonstrating fiducial misalignment (green contour).

**Figure 2:**
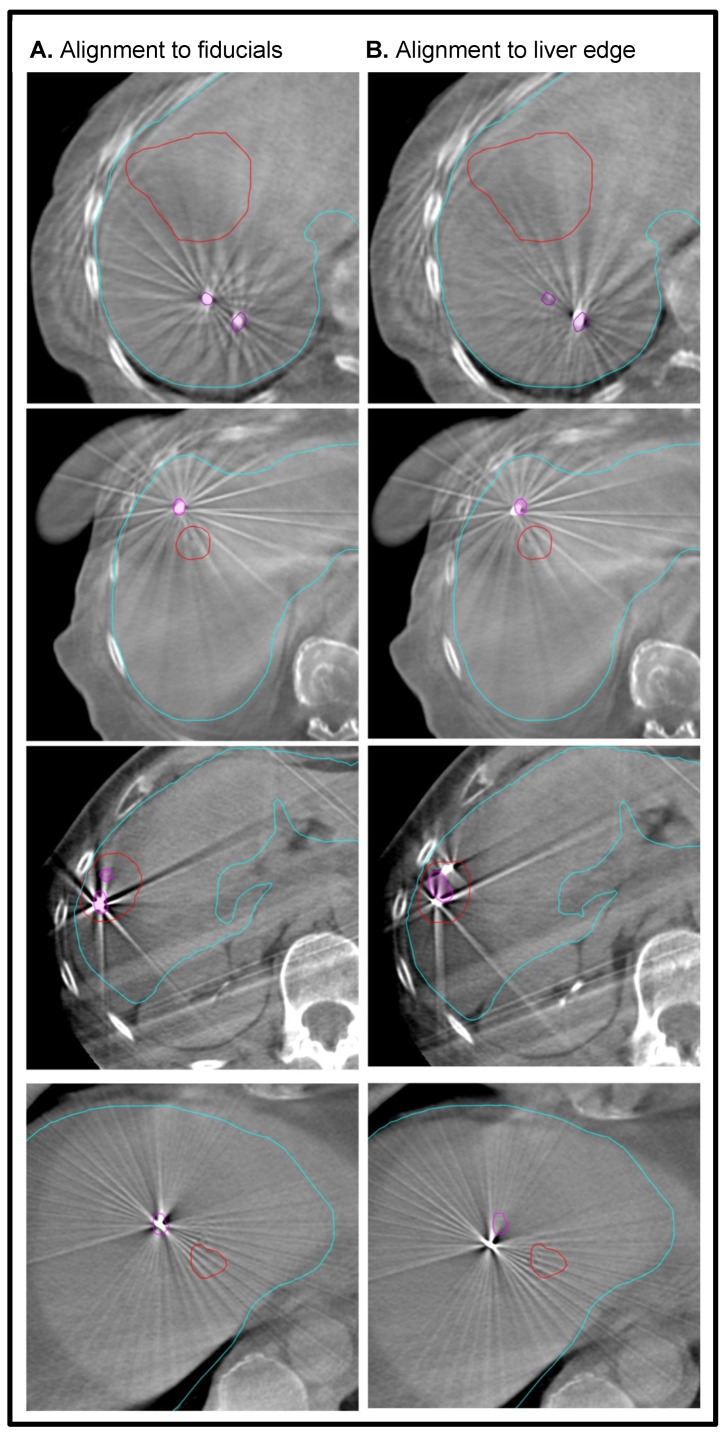
Representative images of alignment concordance from four patients. The left column (**A**) shows alignments based on fiducial position, and the right column (**B**) shows alignment based on liver surface. The top two rows represent good agreement between fiducial and liver surface alignment, while the bottom two rows represent poorer agreement. Note that, in some cases, alignment based on liver surface also involved adjusting the superior–inferior position. Contours: fiducials (magenta), GTV (red), and liver edge (cyan).

To quantify the accuracy of fiducial marker-based localization, seven observers (Radiation Oncology physicians) retrospectively analyzed the CBCT scans acquired before each fraction. The Aria Eclipse platform was used for target localization through viewing CBCT scans in the offline review mode. For each CBCT, two alignments were performed (Figure 1). First, the images were aligned to the liver edge in the region of the GTV. Second, the CBCT scan was aligned to the fiducial markers, using the GTV and fiducial marker contours as a guide. Figure 2 demonstrates examples of concordance between alignment to the liver edge or fiducial markers. In each case, the alignment was reset- to the acquisition position prior to matching. For each alignment, the anterior–posterior (AP), superior–inferior (SI), and lateral or left–right (LR) position was recorded. The mean position across all observers of the CBCT alignments based on fiducial markers was considered the ground truth for subsequent analysis.

The error in each observation was calculated as the shift relative to the ground truth alignment. The uncertainty for both fiducial and liver edge alignment was calculated using inter-observer variability (i.e., the standard deviation (SD) of the observed positions). Descriptive statistics were used to evaluate the impact of the target size, distance to the closest liver surface, and distance to the liver dome caused by the shift magnitude. Patients were divided into two groups based on the median of each clinical factor, and the means of these groups were compared using a *t*-test.

## 3. Results

### 3.1. Patient, Tumor, and Treatment Characteristics

The tumor characteristics for 19 patients are summarized in Table 1. Of the 24 lesions treated with SBRT, 20 were liver metastases (83%), with the most common primary metastases being colorectal cancer in 9 patients (45%). The remaining four lesions were primary liver cancers (17%). The median treatment dose was 5000 cGy (range 3000–5400 cGy) delivered in 3–5 fractions. More than half of the lesions were located in segment VII or VIII (58%). The median GTV was 11.5 cm^3^ (range 0.2–291 cm^3^) and median PTV was 38.5 cm^3^ (range 3.8–453 cm^3^). All patients had at least one radio-opaque marker located near the liver lesion, with a median of 2 fiducials (range 2–4) being placed. In two cases, surgical clips or lipiodol were used for localization.

### 3.2. Inter-Observer Variability

**Figure 3:**
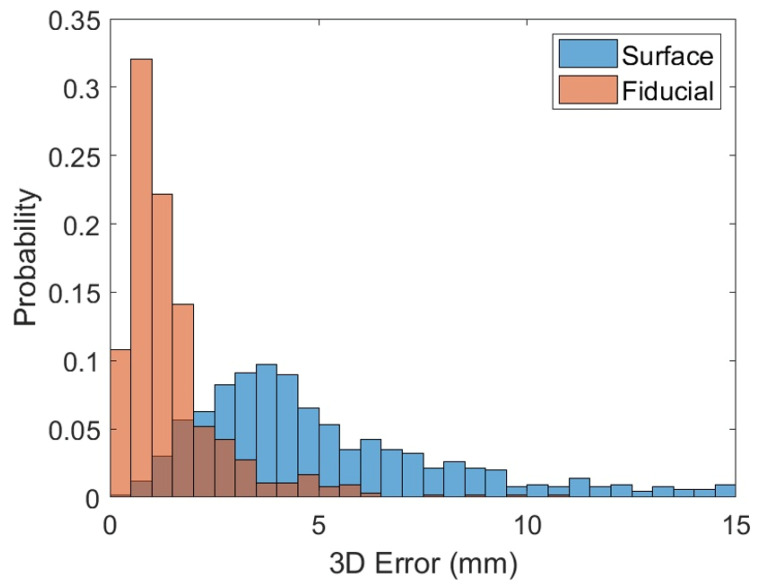
Histogram showing the distribution of all errors. Shown here is the distribution of errors from all patients and observers when aligning to either the liver surface (blue) or fiducials (red). Results are shown as 3D/Cartesian errors, which combine the total error in the AP, SI, and LR directions. The probability is normalized such that the sum of each histogram is 1.

The mean absolute error and uncertainty determined by seven observers using the liver surface or fiducial markers for patient realignment are shown in Table 2. For the Cartesian shift, the mean absolute error was over 3.5 times greater and the mean uncertainty was 2.5 times as high when aligning to the liver surface compared to fiducial markers. For each observer, an individual error of 5 mm or greater was observed 50% of the time when aligning to the liver surface versus 5% of the time when aligning to fiducial markers. A histogram of the errors for all observations using the liver surface versus fiducial markers is shown in Figure 3. In addition to the higher average error when aligning to the liver surface, the error distribution has a long tail, and in a small number of cases very large errors were observed.

### 3.3. Impact of Tumor Parameters on Magnitude of Shifts

In order to identify patients that may especially benefit from fiducial placement, several patient-specific parameters were analyzed for correlation with the surface-based set-up error. These parameters included the GTV volume, distance to closest liver surface, and distance from the superior tumor margin to the liver dome. These results are shown in Table 3; tumors with a greater distance to the liver dome had a higher average error when aligning to the liver surface.

## 4. Discussion

This is the first study specifically designed to determine the utility of fiducial-based alignment compared to liver edge-based alignment, and the inter-observer reliability of these alignments were investigated. We discovered that treatment set-up without fiducial markers in liver SBRT leads to a significantly greater mean positional error of 5.3 mm compared to that of 1.5 mm with a fiducial-based set-up. Inter-observer variability between seven observers was significantly higher when aligning to the liver edge versus to fiducial markers, specifically, 4.5 mm versus 1.8 mm, respectively, suggesting that fiducial markers allow more accurate and reproducible liver SBRT delivery.

Implanted fiducial markers have demonstrated trends of improved local control [9] and superiority in guiding treatment when compared to other alternative set-up strategies such as no correction, alignment to bony anatomy, or alignment to the diaphragm [6,7,8]. Mathew et al. evaluated the long-term outcomes in localized HCC patients who were not candidates of other liver-directed treatments and received SBRT. Upn univariate analysis, the use of fiducial markers had a lower likelihood of local recurrence. However, only a small proportion of patients in this study received fiducial implants [9]. Wunderink et al. determined day-to-day displacements using marker guidance versus conventional surrogates for liver SBRT. The authors found that when the markers were implanted near the tumor, random and systematic error were 0.9 mm and 0.4 mm, respectively. Using conventional set-up methods using an uncorrected frame-based set-up, bony anatomy and diaphragm tip cranial caudal registration had inferior residual set-up errors for this patient group (1.4 mm< o < 2.8 mm; 2.6 mm < E < 5.1 mm) [8]. Similarly, Seppenwoolde et al. compared the accuracy of liver tumor position using marker guidance versus other surrogates and showed that marker-guided treatment set-up accuracy decreases significantly (*p* < 0.001) with increased distance between the marker and tumor. The random and systematic prediction error SDs at a marker tumor distance of 2 cm were 1.0 mm and 1.2 mm, respectively [7]. Finally, Bertholet et al. in a comparable treatment setting identified a marker-based set-up error of 2.2 mm without and 1.3 mm with translational correction [6]. The largest set-up error of 5.8 mm resulting from using vertebral alignment.

In this study, we utilized the closest fiducial from the tumor with an average distance of 0.4 cm and found significantly decreased random and systematic errors of 1.5 mm and 1.8 mm when compared to those of a liver surface surrogate whereby the random and systematic errors were 5.3 mm and 4.5 mm, respectively, similarly to those of some of the conventional surrogates used. We identified that tumors 3 cm or farther from the liver dome had higher mean errors when aligned without fiducials (4.8 cm vs. 4.4 cm, *p* = 0.003). These patients may potentially derive additional benefit from fiducial marker placement as the liver dome may serve as a reproducible landmark for daily set--up whereas other regions of the liver may be more difficult to align on CBTC scans due to the presence of more deformation and the lack of identifiable landmarks.

Although data suggest that fiducial markers may provide superior localization and trends towards improved local control, the procedure of implanting fiducial markers is not without risk. In addition to the risks inherent in a minimally invasive surgical procedure, such as bleeding, infection, or damage to nearby structures, patients with hepatocellular carcinoma or liver metastasis may be particularly vulnerable given their compromised liver function and potentially higher susceptibility to bleeding post-implantation. Ideal placement of fiducials is also crucial and may be impacted by the experience of the operator, tumor location, nearby vasculature, and/or imaging capability [8].

Potential limitations of this study include the limited sample size which may not be representative of other patient populations treated at other institutions. We focused on a single representative fiducial marker closest to the target for our measurements and did not assess shifts based on additional markers. Additional limitations include the precise placement of fiducial markers and random error, such as day-to-day motion which cannot be easily predicted or controlled. The differential fiducial-to-tumor motion and tumor deformations were not considered in our analysis as these data were unavailable. Lastly, our study focused on variation and did not investigate the impact on clinical outcome.

## 5. Conclusions

In conclusion, aligning to the liver edge resulted in significantly increased inter-observer variability in daily set-up and greater error in set-up shifts when compared to alignment to implanted fiducial markers. Our data support use of fiducial markers for safe and accurate liver SBRT delivery. Further work is needed to identify a subset of patients that can be treated accurately with an omission of fiducials or through using magnetic resonance (MR)-based linear accelerators that can better localize (a) liver tumor(s).

## Figures and Tables

**Table 1:** Patient tumor and treatment characteristics (*n* = 24).

Characteristics	*n* (%)
Disease type	
Primary liver cancer	4 (17)
Liver metastasis	20 (83)
Histology of metastases	
Colorectal adenocarcinoma	9
Neuroendocrine	1
Esophageal adenocarcinoma	1
Pancreaticobiliary	1
Breast adenocarcinoma	1
Sarcoma	1
GIST	2
Ovarian	2
Small bowel	2
Lesion segment	
II	4 (17)
Iva	2 (8)
V	2 (8)
VI	2 (8)
VII	7 (29)
VIII	7 (29)
SBRT dose, cGy	
Median	5000
Range	3000–5400
No. of fractions	
Median	3
Range	3–5
GTV volume, cm^3^	
Median	11.5
Range	0.2–291
PTV volume, cm^3^	
Median	38.5
Range	3.8–453
Motion management	
Abdominal compression	11 (46)
Respiratory gating	13 (54)
No. of fiducials	
Median	2
Range	2–4

Abbreviations: cGy = centigray; GIST = gastrointestinal stromal tumor; GTV = gross tumor volume; No. = number; PTV = planning target volume; SBRT = stereotactic body radiotherapy.

**Table 2:** Inter-observer variability.

	AP	SI	LR	Cartesian
Liver Surface				
Mean error (mm)	2.6	2.8	2.5	5.3
Mean uncertainty (mm)	2.3	2.8	2.2	4.5
Fiducial Markers				
Mean error (mm)	0.7	0.9	0.6	1.5
Mean uncertainty (mm)	1.0	1.1	0.8	1.8

Abbreviations: AP = anterior–posterior, LR = left–right, mm = millimeter, SI = superior–inferior.

**Table 3:** Effect of clinical factors on surface-based alignment error.

	Surface-Based Alignment Error	*p*
GTV volume		0.16
<11.5 cc	5.6 ± 2.6 mm	
>11.5 cc	4.9 ± 2.0 mm	
Distance to liver surface		0.13
0 cm	5.5 ± 2.7 mm	
>0 cm	4.7 ± 1.4 mm	
Distance to liver dome		0.003
<3.3 cm	4.6 ± 1.4 mm	
>3.3 cm	6.0 ± 2.9 mm	

## Data Availability

Research data are stored in an institutional repository and will be shared upon request to the corresponding author.

## References

[1] Jackson W.C., Tao Y., Mendiratta-Lala M., Bazzi L., Wahl D.R., Schipper M.J., Feng M., Cuneo K.C., Lawrence T.S., Owen D. (2018). Comparison of Stereotactic Body Radiation Therapy and Radiofrequency Ablation in the Treatment of Intrahepatic Metastases. Int. J. Radiat. Oncol. Biol. Phys..

[2] Lee M.T., Kim J.J., Dinniwell R., Brierley J., Lockwood G., Wong R., Cummings B., Ringash J., Tse R.V., Knox J.J. (2009). Phase I study of individualized stereotactic body radiotherapy of liver metastases. J. Clin. Oncol..

[3] Rusthoven K.E., Kavanagh B.D., Cardenes H., Stieber V.W., Burri S.H., Feigenberg S.J., Chidel M.A., Pugh T.J., Franklin W., Kane M. (2009). Multi-institutional phase I/II trial of stereotactic body radiation therapy for liver metastases. J. Clin. Oncol..

[4] Wahl D.R., Stenmark M.H., Tao Y., Pollom E.L., Caoili E.M., Lawrence T.S., Schipper M.J., Feng M. (2016). Outcomes after Stereotactic Body Radiotherapy or Radiofrequency Ablation for Hepatocellular Carcinoma. J. Clin. Oncol..

[5] Goodman K.A., Kavanagh B.D. (2017). Stereotactic Body Radiotherapy for Liver Metastases. Semin. Radiat. Oncol..

[6] Bertholet J., Worm E., Hoyer M., Poulsen P. (2017). Cone beam CT-based set-up strategies with and without rotational correction for stereotactic body radiation therapy in the liver. Acta Oncol..

[7] Seppenwoolde Y., Wunderink W., Wunderink-van Veen S.R., Storchi P., Mendez Romero A., Heijmen B.J. (2011). Treatment precision of image-guided liver SBRT using implanted fiducial markers depends on marker-tumour distance. Phys. Med. Biol..

[8] Wunderink W., Mendez Romero A., Seppenwoolde Y., de Boer H., Levendag P., Heijmen B. (2010). Potentials and limitations of guiding liver stereotactic body radiation therapy set-up on liver-implanted fiducial markers. Int. J. Radiat. Oncol. Biol. Phys..

[9] Mathew A.S., Atenafu E.G., Owen D., Maurino C., Brade A., Brierley J., Dinniwell R., Kim J., Cho C., Ringash J. (2020). Long term outcomes of stereotactic body radiation therapy for hepatocellular carcinoma without macrovascular invasion. Eur. J. Cancer.

